# Environmental Conditions Determine the Course and Outcome of Phytoplankton Chytridiomycosis

**DOI:** 10.1371/journal.pone.0145559

**Published:** 2015-12-29

**Authors:** Thomas Rohrlack, Sigrid Haande, Åge Molversmyr, Marcia Kyle

**Affiliations:** 1 Norwegian University of Life Sciences, Institute for Environmental Studies, Postbox 5003, NO-1432 Ås, Norway; 2 Norwegian Institute for Water Research, Gaustadalléen 21, NO-0349 Oslo, Norway; 3 International Research Institute of Stavanger, Professor Olav Hanssens vei 15, NO-4021 Stavanger, Norway; INRA, FRANCE

## Abstract

Chytrid fungi are highly potent parasites of phytoplankton. They are thought to force phytoplankton organisms into an evolutionary arms race with high population diversity as the outcome. The underlying selection regime is known as Red Queen dynamics. However, our study suggests a more complex picture for chytrid parasitism in the cyanobacterium *Planktothrix*. Laboratory experiments identified a “cold thermal refuge”, inside which *Planktothrix* can grow without chytrid infection. A field study in two Norwegian lakes underlined the ecological significance of this finding. The study utilized sediment DNA as a biological archive in combination with existing monitoring data. In one lake, temperature and light conditions forced *Planktothrix* outside the thermal refuge for most of the growing season. This probably resulted in Red Queen dynamics as suggested by a high parasitic pressure exerted by chytrids, an increase in *Planktothrix* genotype diversity over time, and a correlation between *Planktothrix* genotype diversity and duration of bloom events. In the second lake, a colder climate allowed *Planktothrix* to largely stay inside the thermal refuge. The parasitic pressure exerted by chytrids and *Planktothrix* genotype diversity remained low, indicating that *Planktothrix* successfully evaded the Red Queen dynamics. Episodic *Planktothrix* blooms were observed during spring and autumn circulation, in the metalimnion or under the ice. Interestingly, both lakes were dominated by the same or related *Planktothrix* genotypes. Taken together, our data suggest that, depending on environmental conditions, chytrid parasitism can impose distinct selection regimes on conspecific phytoplankton populations with similar genotype composition, causing these populations to behave and perhaps to evolve differently.

## Introduction

Phytoplankton are at the base of aquatic food webs and any disturbance of this group can have ripple effects throughout the aquatic community. Traditionally, growth resources, sedimentation and herbivorous grazing have been seen as the main drivers of phytoplankton dynamics and composition [1,2]. However, recent molecular surveys underlined the importance of parasitism as a loss process [3,4]. In particular parasitism by chytrid fungi, known as chytridiomycosis, has been suggested to significantly impact phytoplankton dynamics and composition [5,6]. The probability of chytrid infection increases with the abundance of phytoplankton hosts [7]. Therefore, chytrids are efficient antagonists of bloom forming phytoplankton, including cyanobacteria [8,9] and diatoms [10,11].

According to research on the diatom *Asterionella formosa*, phytoplankton chytridiomycosis may result in a coevolutionary arms race with Red Queen dynamics [12,13]. Typical for the Red Queen dynamics is a regime of time-lagged, negative frequency-dependent selection that leads to diversification of host populations [12,13]. The reason for this is that a high diversity makes it more difficult for a parasite to efficiently exploit its host. Host diversification thus temporarily reduces the relative fitness of a parasite until it manages to adapt. Therefore, the Red Queen dynamics should yield phytoplankton populations that are characterized by high genotype diversity and high genotype turnover [10,14].

Studies on the filamentous cyanobacterium *Planktothrix* support these predictions and suggest a possible mechanism for the Red Queen dynamics. Chytrids can inflict considerable mortality on *Planktothrix* [9,15]. *Planktothrix* genotypes, on the other hand, can produce distinct sets of bioactive oligopeptides that contribute to a genotype-specific anti-chytrid defensive system [15,16]. Chytrids can more readily adapt to the oligopeptides of a specific *Planktothrix* genotype [16]. This adaptation exerts selective pressure on the respective *Planktothrix* genotype, while it may also make it more difficult for the respective parasites to exploit *Planktothrix* genotypes with other sets of oligopeptides [15,16]. The resulting evolutionary arms race should, according to the Red Queen hypothesis, lead to a subdivision of *Planktothrix* populations into coexisting genotypes with distinct sets of oligopeptides. This is in agreement with most field observations [15,17].

Yet, results of a recent field study are difficult to reconcile with the Red Queen hypothesis [18]. Here, host diversity was seen to decrease over time until a single *Planktothrix* genotype dominated the local *Planktothrix* population with dense episodic blooms for at least 15 years. Although chytrids capable of infecting this genotype were present, they failed to control it efficiently. This suggests that *Planktothrix* can escape chytridiomycosis and in turn evade the Red Queen dynamics.

Research has shown that chytrids infecting phytoplankton have narrower temperature tolerance ranges than their hosts. This results in thermal refuges that allow the host to grow without being infected by chytrids [7,19]. Utilizing this finding and to explain the contrasting observations concerning the impact of chytrids on *Planktothrix* populations, we propose the occurrence of two selection regimes in phytoplankton chytridiomycosis, corresponding to a life outside and to a life inside thermal refuges. The first leads to Red Queen dynamics, while the latter allows the host to escape this fate. We further suggest that, depending on local climatic conditions, a given host species can be subjected to either of these selection regimes, resulting in conspecific populations that differ considerably in their properties.

We tested these hypotheses using populations of *Planktothrix* in two lakes in Southern Norway as study objects. The lakes had the same trophic state and offered *Planktothrix* similar access to important nutrients. Yet, located in different climatic zones, one lake allowed *Planktothrix* to permanently live inside a thermal refuge, while in the other lake *Planktothrix* was forced out of such refuge for most of the growing season.

Southern Norway is particularly suited for this type of study, since it offers different climatic zones in close proximity to each other. In addition, the makeup of local *Planktothrix* populations is simple and well-studied. Populations comprise combinations of only four major genotypes [17,20–22] that have been studied genetically [23]. They produce distinct sets of oligopeptides with different activities against local chytrid genotypes [15]. The four *Planktothrix* genotypes were earlier called Cht1, Cht5, Cht7 and Cht9 and we chose to keep these names to maintain consistency. *Planktothrix* populations of Southern Norway are not separated from each other, but instead seem to exchange genotypes easily [15,21,24]. The same is true for *Planktothrix*-infecting parasitic chytrids in this region [15]. This creates a network of host and parasite populations that easily can increase diversity by assimilating genotypes from neighboring populations.

## Material and Methods

### Thermal refuges of local *Planktothrix* genotypes

A thermal refuge is defined as a temperature range that allows a host to grow without being infected by a parasite. Phytoplankton species can have two such refuges [19], a “cold refuge” that spans from the lower temperature limit for host growth to the lower limit for chytrid infection, and a “warm refuge”, spanning from the upper temperature limit for chytrid infection to the upper temperature limit for host growth. The actual location of these refuges on the temperature scale may vary from host to host. We thus started our study by identifying thermal refuges of local *Planktothrix* genotypes. The genotypes Cht1, Cht5, Cht7 and Cht9 differ in up to 17 percent of their coding genes, with Cht1 and Cht9 being the most distantly related among the four genotypes [23]. To reflect this genetic diversity, we utilized four laboratory isolates, two representing Cht1 (NIVA-CYA98, NIVA-CYA406) and two representing Cht9 (NIVA-CYA278, NIVA-CYA405), when determining the thermal refuges of local *Planktothrix* genotypes. The isolates were cultured under sterile conditions in 250 ml flasks with continuous aeration by diluting them to an optical density of 0.062 (5 cm cuvette, 800 nm) every other day. BG11 served as culture medium. Cultures received constant light at a photon flux density of 20 μmol m^-2^ s^-1^. The culture temperature was 6, 9, 12, 15, 18, or 21 ± 0.2°C. When all cultures had reached steady state, their specific growth rates were determined for a period of 10 days, using measurements of optical density at 800 nm as basis.

From the various chytrid isolates that were established from *Planktothrix*-dominated lakes in Southern Norway, we selected the two with highest genetic distance to study the effect of temperature on chytrid infectivity. This was done to ensure that results reflect the diversity of local *Planktothrix* infecting chytrids. A detailed description of the chytrid isolates Chy-Kol2008 and Chy-Lys2009 as well as of their host ranges is given in an earlier publication [15]. Briefly, Chy-Kol2008 and Chy-Lys2009 were isolated from Lakes Kolbotnvannet and Lyseren (both situated in Southern Norway) in 2008 and 2009, respectively. Both represent chytrid genotypes that occur in *Planktothrix* dominated lakes throughout Southern Norway. Both utilize species of the genus *Planktothrix* as their only hosts. Other filamentous cyanobacteria or dead organic matter are not accepted as host/food source. Morphology and life cycle identify Chy-Kol2008 and Chy-Lys2009 as *Rhizophidium megarrhizum* Sparrow 1943. Sequence identity between both isolates is 98.2% in the 28S region and 86.2% in the ITS region (see [15] for sequence information and phylogenetic analysis).

Chy-Kol2008 and Chy-Lys2009 were cultured as described earlier [16]. Chytrids propagate with zoospores that are formed in epiphytic sporangia [8]. Chy-Kol2008 and Chy-Lys2009 zoospore suspensions were produced by filtering chytrid cultures over 10 μm gauze. The density of zoospore suspensions was then determined with a hemocytometer.

Infection experiments utilized material from the above described steady state *Planktothrix* cultures of isolates NIVA-CYA98, NIVA-CYA406, NIVA-CYA278, and NIVA-CYA405. The experiments were conducted in 24-well polystyrene microtiterplates. *Planktothrix* and zoospore suspensions were individually mixed into each well at a final volume of 2 ml. The final *Planktothrix* density corresponded to an optical density of 0.03, while that of chytrid zoospores was 50000 ml^-1^. The microtiterplates were kept at the temperature to which the respective *Planktothrix* cultures were acclimated, i.e., at 6, 9, 12, 15, 18, or 21 ± 0.2°C. Light conditions were identical to those described above. After 48 hours, the prevalence of chytrid infection, defined as % host filaments infected, was determined by light microscopic inspection of 100 *Planktothrix* filaments per replicate. Only filaments carrying at least one epiphytic sporangium were counted as successfully infected. Here it must be considered that, as indicated by its name, rhizoids of the species *R*. *megarrhizum* can be very long, often spanning entire *Planktothrix* filaments. % infected filaments is therefore a reasonable way to express prevalence of chytrid infection in *Planktothrix*. All tests were run under sterile conditions in quadruplicates.

The relationships between temperature and specific *Planktothrix* growth rate and between temperature and prevalence of chytrid infection were fitted to logarithmic functions. Thermal refuges were located by combining the zero points of both functions. The procedure was repeated for all host-parasite combinations. All organisms used in this activity are available from the Norwegian University for Life Sciences and were originally supplied by the Norwegian Institute for Water Research culture collection of algae.

### Safe zones for *Planktothrix* in Lakes Kolbotnvannet and Hålandsvatnet

The thermal refuges of a given phytoplankton host define safe zones of a lake’s water column, where the host may grow without chytrid infection. However, phytoplankton organisms such as *Planktothrix* also need light to grow. The maximal depth of safe zones is therefore limited to the photic zone depth of a lake. For *Planktothrix* in Lakes Kolbotnvannet and Hålandsvatnet, safe zones were localized on the basis of (1) results of above laboratory tests, (2) the temperature depth profiles for April-October that were calculated as average for the years 2000–2013, and (3) the average photic zone depths calculated for the years 2000–2013. Photic zone depths were calculated on the basis of secchi depth measurements using a power function [25]. Characteristics of Lakes Kolbotnvannet and Hålandsvatnet are compiled in Table 1.

**Table 1:** Characteristics of Lakes Kolbotnvannet and Hålandsvatnet.

	Lake Hålandsvatnet	Lake Kolbotnvannet
Location	58°58'19"N, 5°38'26"E, about 6 km west of the city of Stavanger in close proximity to the Atlantic Ocean	59°48'15"N, 10°47'56"E, about 10 km south of the city of Oslo
Climate zone	Oceanic climate zone	Boral climate zone
Mean temperature and wind speed^1^	8.47°C, 4.59 m s^-1^	6.30°C, 2.58 m s^-1^
Area, mean and maximum depth^2^	1.1 km^2^, 9.4 m, 25 m	0.3 km^2^, 10.3 m, 18 m
Catchment area^2^	7.9 km^2^	3.0 km^2^
Mean total phosphorus and total nitrogen concentration^2^	29.1 μg L^-1^, 1400 μg L^-1^	31.4 μg L^-1^, 600 μg L^-1^
Mean chlorophyll concentration^2^	17.9 μg L^-1^	19.7 μg L^-1^

^1^Temperature and wind data supplied by the Norwegian Meteorological Institute via the web portal eKlima.no, for Ås and Sola weather stations for the years 2000–2013.

^2^Mean values calculated for period 2000–2013. Raw data are from local monitoring projects and are given in [26] for Lake Kolbotnvannet and in [27–29] for Lake Hålandsvatnet. Please refer to the web pages www.niva.no and http://www.vannportalen.no/vannregioner/rogaland/vannomrader/jaren/overvaking/halandsvatnet/ to access these reports.

Temperature profiles and secchi depths came from local monitoring projects, which were independent of our study. The same monitoring projects also provided *Planktothrix* biovolume concentrations for the layer 0–4 m in both lakes as well as depth distributions of *Planktothrix* in Lake Kolbotnvannet during the summer of 2005. These were determined using a fluorescence probe (bbe Moldaenke GmbH, Schwentinental, Germany) calibrated to the *Planktothrix* genotype Cht1. Detailed information on the monitoring projects can be found elsewhere [26,27].

### *Planktothrix* genotype composition and parasitic pressure exerted by chytrids

This part of the study utilized sedimentary DNA to act as biological archive covering the years 2000–2013. Sediment cores were taken at the deepest points of Lakes Kolbotnvannet and Hålandsvatnet using a gravity corer. All cores were cut into 1 cm slices throughout. Samples for dating were dried at 60°C and subsequently analyzed for ^137^Cs content using a Sodium-Iodine detector (Wallac 1480 Wizard 3" gamma counter, PerkinElmer, Oslo, Norway). The ^137^Cs peak core depth was converted to age by assuming that it represented April 1986, the date of the Chernobyl nuclear accident. In Lake Hålandsvatnet, dating was further facilitated by the distinct colorations of spring and summer depositions, giving the top of sediment cores clearly visible “annual rings”.

Sediment DNA was extracted using the PowerSoil^®^ DNA Isolation kit (MoBio Laboratories, Inc., Carlsbad, CA USA; cat.no. 12888) as described earlier [18,30]. The *Planktothrix* genotypes Cht1, Cht5, Cht7 and Cht9 were individually quantified using specific qPCR methods that were described by Kyle and coworkers [18]. Specificity was verified in this earlier study. This was facilitated by the availability of eight *Planktothrix* genome sequences [23] and access to many partially sequenced *Planktothrix* isolates [20]. *Planktothrix* infecting chytrids were quantified as a group. The respective qPCR method was described earlier [18]. Its specificity was verified in this earlier study using cultures of *Planktothrix* infecting chytrids from Southern Norway, including Chy-Kol2008 and Chy-Lys2009, and publically available sequences from chytrid isolates with phytoplankton hosts other than *Planktothrix*. Sediment DNA undergoes degradation. The qPCR method accounted for this by using primers designed to amplify short DNA fragments of similar length for all organisms of interest. We further reduced the impact of DNA degradation by relying on sediment DNA not older than 15 years, by choosing lakes with a high sedimentation rate, i.e., lakes with a rapid burial of newly deposited DNA, and by working in lakes that experience long periods with anoxic conditions in the hypolimnion [30].

The abundances of *Planktothrix* genotypes and *Planktothrix*-specific chytrids were calculated as ng DNA of the amplified region per g organic matter in the sediment. The content of organic matter in the sediment was determined for each sample as loss of ignition at 550°C. The parasitic pressure that chytrids exert on a given *Planktothrix* population was estimated as the ratio of the cumulative abundance of *Planktothrix* genotypes and the abundance of *Planktothrix*-specific chytrids. This approach builds on the finding of our earlier study that chytrids infecting *Planktothrix* are obligate parasites that have no alternative hosts or food sources [15].

### Ethics statement

According to Norwegian law, sampling of lakes and the usage of result of field studies for research purposes do not require permission as long as the studies are conducted outside protected environments and do not involve humans or protected organisms. Therefore, our study did not require any permission.

## Results

### The thermal refuges of local *Planktothrix* genotypes

**Fig 1:**
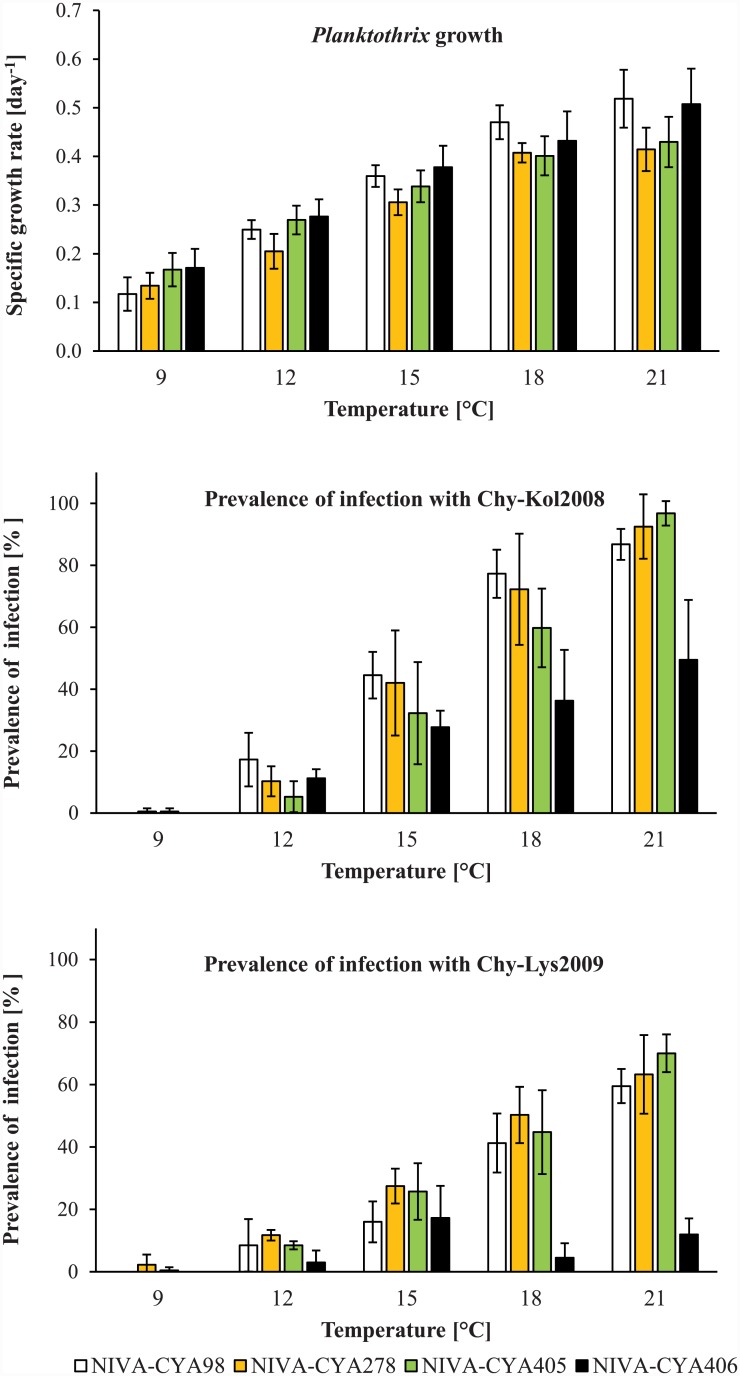
Effect of temperature on growth and chytrid infectivity in *Planktothrix*. Upper diagram—specific growth rate of four *Planktothrix* isolates at five temperatures. The columns represent mean values of five measurements with the respective standard deviations. Middle diagram—infectivity of chytrid isolate Chy-Kol2008 in the same four *Planktothrix* isolates at the same temperatures. Infectivity was measured as prevalence of infection in *Planktothrix* after 2 days of exposure to chytrid Chy-Kol2008. The columns represent mean values of four measurements with the respective standard deviations. Lower diagram—same as before but with chytrid Chy-Lys2009 as parasite.

At 6°C, all *Planktothrix* genotypes grew at rates that could not be measured reliably with our method. This made it impossible to determine the growth rate of *Planktothrix* and to run infection experiments at 6°C. The temperature was therefore excluded for the subsequent regression analyses. Above 6°C, the specific growth rate of the four *Planktothrix* isolates increased with temperature (Fig 1, upper diagram) and both parameters were tightly correlated (Table 2). The lower limit for *Planktothrix* growth was mathematically estimated to be 5.2–7.1°C, depending on the *Planktothrix* isolate that was tested (Table 2). No upper temperature limit for growth was found within the temperature range that was studied here. In infection experiments with chytrids Chy-Kol2008 and Chy-Lys2009, the prevalence of infection typically increased with temperature (Fig 1, middle and lower diagram). The lower limit for chytrid infection was mathematically estimated to be 9.5–11.6°C, once again depending on the *Planktothrix* isolate that was tested (Table 2). No upper temperature limit for chytrid infection was found within the temperature range that was studied here. According to these data, the “cold thermal refuge” of *Planktothrix* in the study area was estimated to the temperature range 5.2–11.6°C. Local *Planktothrix* and chytrid genotypes seem not to have a “warm thermal refuge” with relevance to Norwegian conditions.

**Table 2:** Results of regression analyses for and thermal refuges of four laboratory *Planktothrix* isolates exposed to two *Planktothrix*-specific chytrid isolates. Regression analyses were done at the 95% level of significance. POI stands for prevalence of infection and n.s. stands for not significant.

Host strain	Relationship between temperature and			Thermal refuges when exposed to	
	host growth (R^2^)	POI with Chy-Kol2008 (R^2^)	POI with Chy-Lys2009 (R^2^)	Chy-Kol2008 [°C]	Chy-Lys2009 [°C]
NIVA-CYA98	0.5*ln(x)-1.0 (0.99)	130*ln(x)-106 (0.98)	94*ln(x)-230 (0.93)	7.1–10.5	7.1–11.6
NIVA-CYA278	0.4*ln(x)-0.7 (0.97)	114*ln(x)-261 (0.94)	74*ln(x)-167 (0.95)	6.4–9.8	6.4–9.5
NIVA-CYA405	0.3*ln(x)-0.5 (0.99)	112*ln(x)-261 (0.88)	80*ln(x)-184 (0.91)	5.2–10.1	5.2–9.9
NIVA-CYA406	0.4*ln(x)-0.7 (1.00)	66*ln(x)-163 (0.99)	n.s.	5.9–10.0	-

### Lake Hålandsvatnet environmental overview

**Fig 2:**
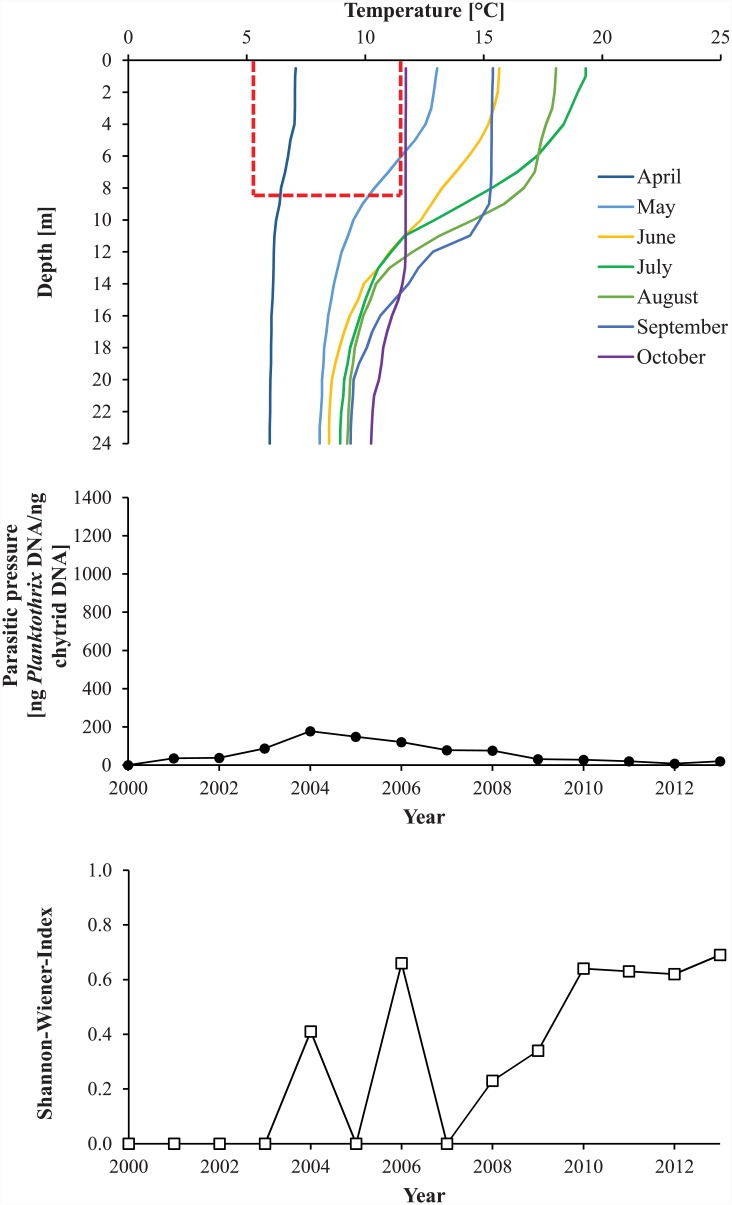
Safe zones, parasitic pressure exerted by chytrids and Shannon-Wiener diversity index in Lake Hålandsvatnet 2000–2013. Upper diagram—average temperature profiles for the months April-October calculated for the years 2000–2013. The years 2000 and 2002–2004 had no monitoring and were therefore not considered. The red box marks the position of the “cold thermal refuge” of *Planktothrix* on the temperature scale and the average photic zone depth on the depth scale. Any part of the water column that falls into this red box is inside a safe zone, allowing *Planktothrix* growth without chytrid infection. Middle diagram—reconstruction of parasitic pressure of chytrids on *Planktothrix* in Lake Hålandsvatnet using sedimentary DNA as biological archive. The parasitic pressure was estimated as ng *Planktothrix* DNA per ng DNA of *Planktothrix*-specific chytrids. The maximal range of the y-axis is the same as in Fig 5 to facilitate comparison of study areas. Lower diagram—reconstruction of Shannon-Wiener diversity index for the local *Planktothrix* population using sedimentary DNA as biological archive. The maximal range of the y-axis is the same as in Fig 5 to facilitate comparison of study areas.

**Fig 3:**
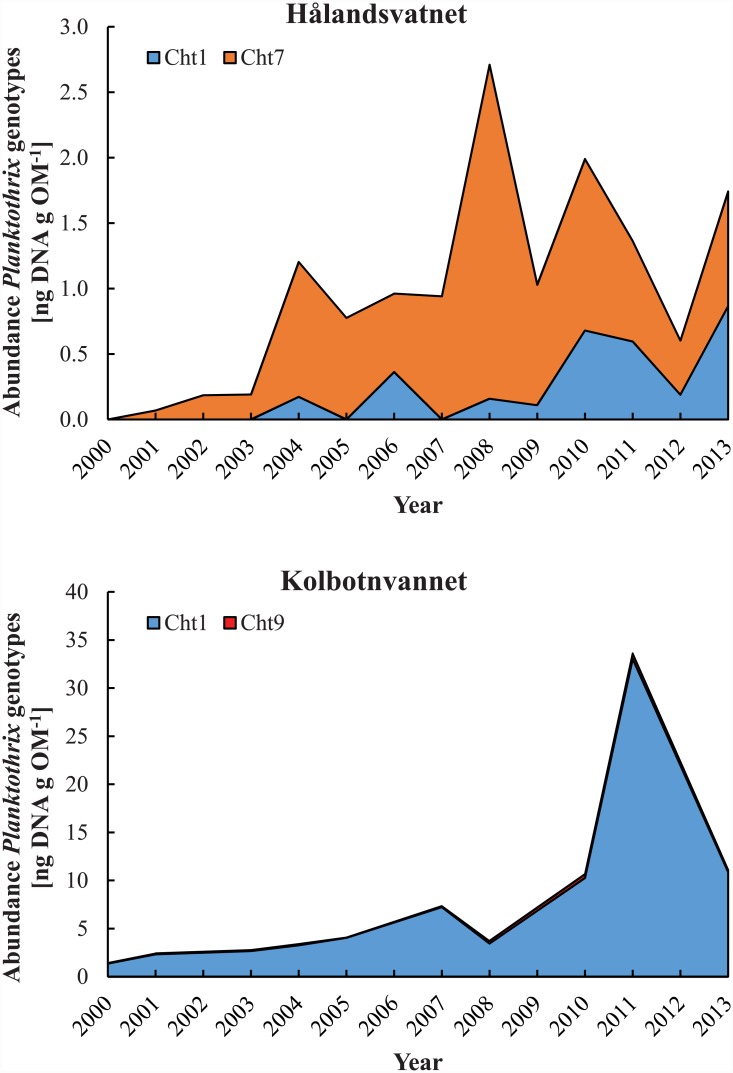
Reconstruction of cumulative *Planktothrix* genotype abundances in Lake Hålandsvatnet (upper diagram) and in Lake Kolbotnvannet (lower diagram) using sedimentary DNA as biological archive.

**Fig 4:**
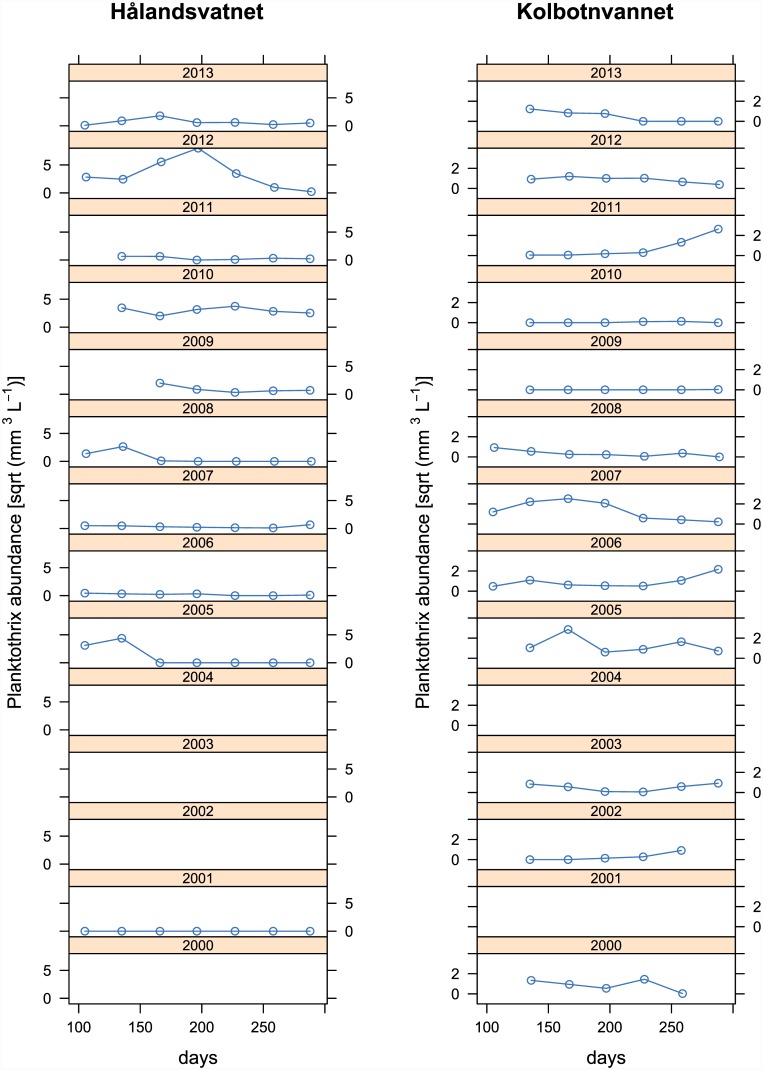
Seasonal dynamics of *Planktothrix* abundance in Lake Hålandsvatnet (left column with diagrams) and in Lake Kolbotnvannet (right column with diagrams) for all years covered by the local monitoring project. Empty diagrams represent years without monitoring.

Due to mild winters and strong winds, the water column of Lake Hålandsvatnet typically reached more than 8°C before stratification set in (Fig 2, upper diagram). The average photic zone depth was 8.4±1.3 m. A typical growing season for *Planktothrix* lasted at least from April to October, when the temperature of the photic zone exceeded the lower limit for *Planktothrix* growth (Fig 2, upper diagram). From June to October, the entire photic zone was too warm to allow *Planktothrix* growth without chytrid infection (Fig 2, upper diagram). This fits with the observation that before the year 2010 *Planktothrix* formed dense blooms in early spring followed by sudden lysis in mid-June [28,29], which would indicate heavy chytrid infection. That chytrids exerted a high parasitic pressure on *Planktothrix* was supported by a low ratio of sediment-derived *Planktothrix* DNA to DNA of *Planktothrix*-specific chytrids throughout the study period (Fig 2, middle diagram). The local *Planktothrix* population was found to be dominated by genotype Cht7 until 2009, although minor amounts of genotype Cht1 were detected between 2004 and 2009 (Fig 3, upper diagram). After 2009, Cht1 gradually became more abundant to reach parity with Cht7 in 2013. No additional genotypes were found. The Shannon-Wiener diversity index was zero until 2004, fluctuated between 2005 and 2007 and subsequently increased until it reached a stable maximum in 2010 (Fig 2, lower diagram). The local monitoring program detected *Planktothrix* for the first time in spring 2005, when it formed a massive bloom near the surface of the lake (Fig 4). This bloom lysed in mid-June and no *Planktothrix* was detected before spring 2006 [28]. The situation repeated itself in 2008 [29]. From 2010, blooms typically lasted much longer, often well into the summer (Fig 4), but were still observed near the surface of the lake. All attempts to explain the first occurrence of a *Planktothrix* bloom in 2005 and bloom patterns in general with abiotic factors failed [27,29]. This, a low abundance of herbivorous zooplankton during bloom events and the often rapid lysis of blooms led to the hypothesis that *Planktothrix* is controlled by parasites or pathogens [28].

### Lake Kolbotnvannet environmental overview

**Fig 5:**
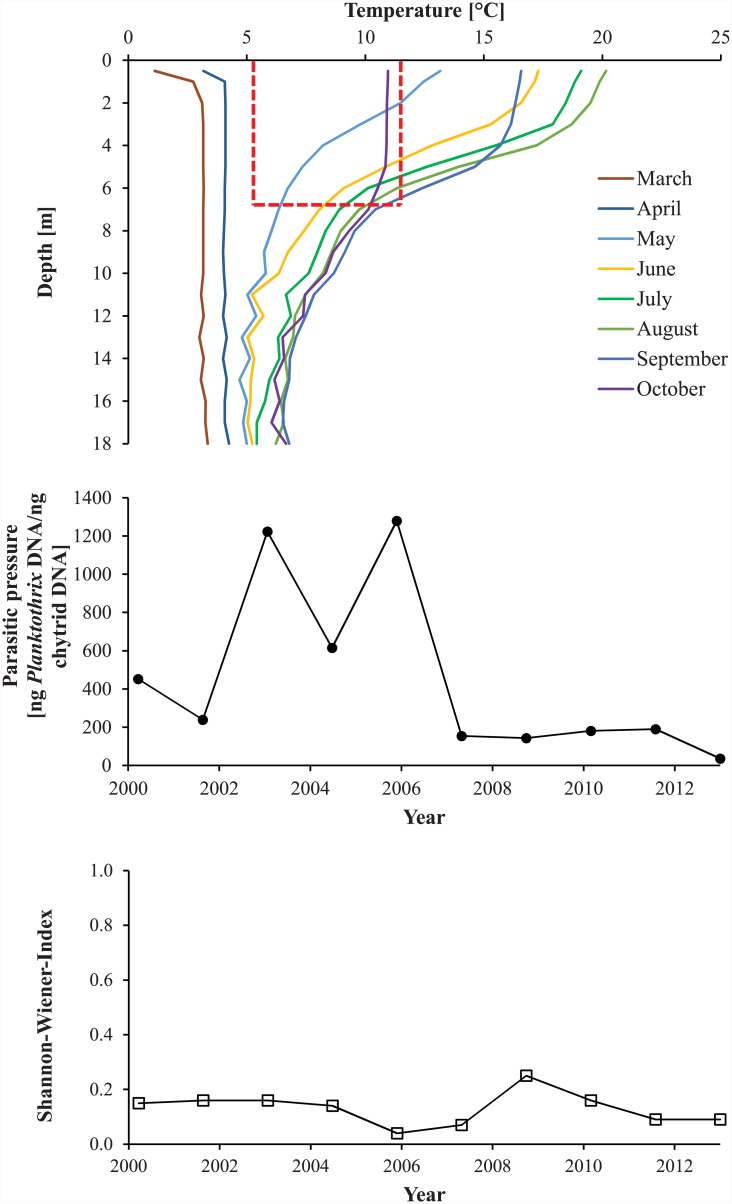
Safe zones, parasitic pressure exerted by chytrids and Shannon-Wiener diversity index in Lake Kolbotnvannet 2000–2013. Upper diagram—average temperature profiles for the months April-October calculated for the years 2000–2013. The year 2001 had no monitoring and was therefore not considered. The red box marks the position of the “cold thermal refuge” of *Planktothrix* on the temperature scale and the average photic zone depth on the depth scale. Any part of the water column that falls into this red box is inside a safe zone, allowing *Planktothrix* growth without chytrid infection. Middle diagram—reconstruction of parasitic pressure of chytrids on *Planktothrix* in Lake Kolbotnvannet using sedimentary DNA as biological archive. The parasitic pressure was estimated as ng *Planktothrix* DNA per ng DNA of *Planktothrix*-specific chytrids. The maximal range of the y-axis is the same as in Fig 2 to facilitate comparison of study areas. Lower diagram—reconstruction of Shannon-Wiener diversity index for the local *Planktothrix* population using sedimentary DNA as biological archive. The maximal range of the y-axis is the same as in Fig 2 to facilitate comparison of study areas.

**Fig 6:**
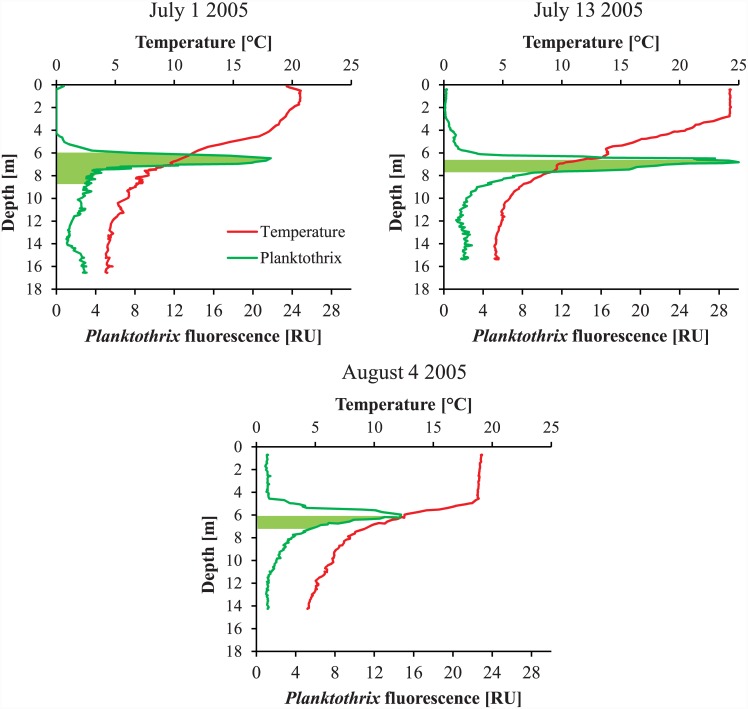
Depth profiles of temperature and *Planktothrix* fluorescence in Lake Kolbotnvannet in the summer of 2005. For each sampling day this part of the *Planktothrix* population that could grow without chytrid infection (safe zone) is shown as light green surface. Please note that the safe zone in this figure was located using the temperature profile and the euphotic zone depth observed during the particular day of sampling, while in Fig 5 the safe zone was located using mean temperature profiles and euphotic zone depths for the entire study period (2000–2013).

Stable thermal stratification typically set in shortly after ice break when the water column had about 5°C (Fig 5, upper diagram). The average photic zone depth was 6.7±0.5 m. The *Planktothrix* growth season usually lasted from May to at least October (Fig 5, upper diagram). Throughout the entire growing season parts of the water column were inside the safe zone that allows *Planktothrix* growth without chytrid infection (Fig 5, upper diagram). An advanced buoyancy regulation system [31] allowed *Planktothrix* to actively move into this safe zone, as was demonstrated for the summer of 2005 (Fig 6). That chytrids exerted a low parasitic pressure on *Planktothrix* was supported by a high ratio of *Planktothrix* DNA to DNA of *Planktothrix*-specific chytrids during the study period (Fig 5, middle diagram). The *Planktothrix* population of Lake Kolbotnvannet was dominated by genotype Cht1 throughout the study period (Fig 3, lower diagram). Small amounts of genotype Cht9 were found as well, but its presence in the local *Planktothrix* population did never surpass 10%. No additional genotypes were found, which is in agreement with results of several strain isolation experiments [20]. The Shannon-Wiener diversity index remained low throughout the study period (Fig 5, lower diagram). The local monitoring program often detected *Planktothrix* throughout the growing season (Fig 4). By visual inspection, *Planktothrix* was typically distributed across the water column in spring and autumn and stratified between 4 and 8 m during summer and under the ice during winter. However, the routine phytoplankton monitoring was based on an integrated sample for 0–4 m. This and seasonal changes in depth distribution resulted in partially distorted measurements of *Planktothrix* abundance. Therefore, the data in Fig 4 must be treated with caution.

## Discussion

### Selection regimes in phytoplankton chytridiomycosis

Data suggest that *Planktothrix* in Lake Hålandsvatnet experienced Red Queen dynamics. This is based on (1) the absence of a safe zone to escape chytrid infection for most of the growing season, (2) a high parasitic pressure exerted by chytrids, (3) rapid lysis of blooms which is typical for chytrid epidemics, (4) an increase in genotype diversity over time and (5) a positive correlation between genotype diversity and duration of bloom events. In contrast, the opposite observations were seen for *Planktothrix* in Lake Kolbotnvannet with (1) persistence of a safe zone, (2) low parasitic pressure exerted by chytrids, (3) long lasting dominance of the same genotype and (4) limited genotype diversity along with a high *Planktothrix* abundance for most of the study period. These findings suggest that *Planktothrix* in Lake Kolbotnvannet could escape the Red Queen arms race with parasitic chytrids. Both *Planktothrix* populations comprised the same (Cht1) or related genotypes (Cht7 and Cht9) [23], but differed in seasonal dynamics, depth distribution and bloom patterns. Hence, our study suggests that chytridiomycosis can impose distinct selection regimes on a given phytoplankton species, causing even genetically similar populations to behave and maybe to evolve differently. Chytridiomycosis may therefore be a force in the phenotypic and genetic diversification in phytoplankton species.

In Lake Kolbotnvannet, *Planktothrix* could escape the Red Queen arms race with parasitic chytrids because the local climate allowed for the formation of a persistent safe zone without chytrid infection. This demonstrates the critical role of local environmental conditions in determining the course and outcome of phytoplankton chytridiomycosis. The success of *Planktothrix* with and without access to a safe zone is, on the other hand, difficult to explain without employment of traits that decrease susceptibility of *Planktothrix* to chytrid infection. These may include the already described chemical defensive system that is based on bioactive oligopeptides [15] and the buoyancy regulation system [31] that allows *Planktothrix* to exploit existing safe zones.

Our work focuses on the cold thermal refuge in *Planktothrix*. However, pioneering work by Bruning [7,32,33] suggests the occurrence of low light and low phosphorus refuges in addition to thermal refuges for diatom hosts. All these refuges have in common that they allow the host to grow without chytrid infection. This may, as shown here for the cold thermal refuge, allow a host to evade the Red Queen dynamics and so to flourish at low genetic diversity for long periods of time.

A second type of refuge may occur when deteriorating environmental conditions force a phytoplankton host into some sort of resting mode without growth. Typical examples are the overwintering of the cyanobacterium *Microcystis* on top of a lake’s sediment [34] or the formation of akinete resting stages in several cyanobacteria. Work by Bruning [32] and that by Gsell and coworkers [19] suggests that under such conditions some chytrids may be unable to sustain their normal life cycle. In some cases, shifting into a resting mode may therefore purge a host population of chytrid infection. In other cases, resting stages may be attacked by specialized chytrids [8]. Regardless, more research is needed to fully explore the importance and consequences of this type of refuge.

### Chytridiomycosis and climate

Recently, Ibelings and coworkers [11] linked the impact of chytridiomycosis on diatom populations to local climate conditions. Our observations in Lakes Kolbotnvannet and Hålandsvatnet support this link. A warmer climate, such as that experienced by Lake Hålandsvatnet, makes it more likely that the photic zone warms up beyond the lower limit for chytrid infection. Beyond that point, any further increase in temperature may amplify the parasitic pressure on *Planktothrix* even more (Fig 1, middle and lower diagram). The likelihood for *Planktothrix* to be forced into Red Queen dynamics may therefore increase with decreasing latitude and increasing altitude. Since Red Queen dynamics drives diversification of *Planktothrix* populations, population diversity should show the same trends. This is supported by a comparison of data from central European [35], alpine [36,37] and Nordic lakes [20].

The impact of chytridiomycosis may also be sensitive to global warming [11]. However, while our laboratory experiments suggest only the presence of a single thermal refuge for *Planktothrix* in Southern Norway, diatoms can have two such refuges, one at low and a second at high temperatures [19]. In addition, the position of the “cold thermal refuge” on the temperature scale differs considerably between *Planktothrix* and diatoms [19]. The impact of global warming on phytoplankton chytridiomycosis is therefore likely to be species-specific.

### Concluding remarks

In 2007, Kagami and coworkers published a list of 100 phytoplankton species with a proven susceptibility to chytrid infection [38]. The list, which probably is far from complete, includes all major groups of phytoplankton, many bloom-forming species and several species with worldwide distribution. Recent molecular surveys verified a high abundance and diversity of parasitic chytrid fungi in freshwater systems [3,4]. And, as demonstrated here, it becomes increasingly clearer that chytridiomycosis can shape phytoplankton populations in a complex and decisive manner. The present study shows, to our knowledge for the first time, that chytrid parasitism in interplay with environmental conditions can impose distinct selection regimes on very similar phytoplankton populations. This finding may help to better understand dynamics and evolution of phytoplankton. Taken together, all available data identify parasitic chytrids as key players of freshwater food webs, about which we still know very little. More studies on phytoplankton chytridiomycosis are therefore urgently needed.
